# Managed alcohol programs for Indigenous Peoples in Canada: A scoping review

**DOI:** 10.1371/journal.pmen.0000651

**Published:** 2026-07-14

**Authors:** Madyson Campbell, Kristen A. Morin, Teresa N. Marsh, Sarah L. Harrison, Danielle S. Labrosse, David C. Marsh

**Affiliations:** 1 Northern Ontario School of Medicine University, Sudbury, Ontario, Canada; 2 Health Science North, Sudbury, Ontario, Canada; 3 Laurentian University, Sudbury, Ontario, Canada; PLOS: Public Library of Science, UNITED KINGDOM OF GREAT BRITAIN AND NORTHERN IRELAND

## Abstract

Managed Alcohol Programs (MAPs), provide regulated access to beverage alcohol alongside housing and health and social supports for individuals with severe AUD and chronic homelessness. While evidence for MAP effectiveness is growing, less is known about their implementation and impact within Indigenous and geographically remote contexts. We conducted a scoping review following PRISMA guidelines evaluate the literature on MAPs operating in Indigenous, northern, rural, and remote communities. A comprehensive search of PubMed, Embase, PsycINFO, and CINAHL was completed in July 2025, supplemented by grey literature and reference list screening. Studies were eligible if they described programs that regulated alcohol consumption for individuals experiencing homelessness and AUD within Indigenous or geographically remote contexts. Two reviewers independently screened articles, and data were extracted using a standardized form. Of 264 records identified, 13 studies met inclusion criteria. Programs were located primarily in Canada, with additional sites in Australia and the United States. Four key themes emerged: (1) integration of Indigenous cultural knowledge and healing practices; (2) housing stability and the creation of “home” within MAPs; (3) reductions in alcohol-related harms, including decreased non-beverage alcohol use and acute healthcare utilization; and (4) barriers and facilitators to implementation. Indigenous-led and culturally grounded MAPs emphasized self-determination, Elders’ involvement, and land- and culture-based healing. Participation in MAPs was consistently associated with improved housing security, safer drinking environments, and reduced health and social harms. However, implementation challenges included stigma, funding instability, and infrastructure limitations in remote settings. MAPs appear to offer a promising, culturally adaptable harm reduction approach for individuals with severe AUD in Indigenous and geographically underserved communities. Indigenous leadership, cultural integration, and community-driven design are critical to program acceptability and sustainability. Further research is needed to evaluate long-term health outcomes and to expand evidence in northern, rural, and remote contexts.

## 1. Introduction

The impact of alcohol consumption along with its harmful effects creates significant challenges for many communities. According to the World Health Organization (WHO), 2.6 million deaths worldwide were attributable to alcohol consumption in 2019, representing 4.7% of all deaths in that year (2024). Its influence on public health contributes to over 200 medical conditions, including infectious diseases, cancers, mental and behavioral disorders, neurological impairments, cardiovascular and gastrointestinal diseases, and injuries [1,2]. Alcohol use poses a substantial burden on population health, highlighting the urgent need for targeted prevention and harm reduction strategies.

Alcohol use disorder (AUD) is defined by the Diagnostic and Statistical Manual of Mental Disorders (DSM-5) as “a problematic pattern of alcohol use leading to clinically significant impairment or distress.” In 2019, an estimated 400 million people aged 15 years and older lived with AUD [2]. Regardless of the high prevalence of AUD, harm reduction treatment options remain limited particularly when compared to the expanding range of harm reduction services available for those who use other substances [3,4]. This disparity in care is especially concerning given the disproportionate impact of AUD on marginalized populations [5,6].

It has been previously shown that despite Indigenous peoples reporting lower alcohol consumption rates [7], the presence of AUD is higher in Indigenous communities compared to the general population [8]. Similarly, individuals living in rural areas have demonstrated higher rates of problematic alcohol use compared to those living in urban areas [9,10]. Beyond these elevated prevalence rates, Indigenous, northern, and rural communities experience disproportionate harms from substance use disorders (SUD) due to a number of factors, including higher rates of poverty, unemployment, and limited access to mental health and addiction services [11,12]. For Indigenous communities, the legacy of colonialism, including cultural disruption and historical trauma, has further compounded these issues, leading to increased vulnerability and harms related to substances [13,14]. Geographic isolation and lack of infrastructure in rural and northern areas can also hinder access to treatment and support services for SUD [15]. Systemic neglect and marginalization contribute to a cycle of adversity that heightens the prevalence and impact of SUD in these communities.

In response to these challenges, managed alcohol programs (MAP) have proven to be a promising intervention aimed at mitigating the adverse effects of AUD. MAP are grounded in the principles of harm-reduction, encompassing strategies that aim to minimize the health, social and economic consequences of a behaviour by prioritizing the reduction of harm without requiring cessation of use [16]. MAP are designed to provide a standardized and supervised intake of beverage alcohol in a supervised and stable setting, often combined with housing and social supports for individuals with severe AUD who experience chronic homelessness [17]. These programs aim to reduce the harms associated with unsupervised drinking and non-beverage alcohol use [18]. They also work to address broader structural challenges faced by individuals with AUD by offering medical care, counseling, and social support tailored to participants’ needs.

Evidence, particularly from Canada, increasingly demonstrates the effectiveness of MAP [19]. However, research on their implementation in Indigenous, northern, and rural communities remain underexplored. Given that these communities may face additional barriers to traditional treatment models, it is essential to evaluate the current research on MAP with factors such as barriers in access to care and cultural differences in mind.

This scoping review aims to review the current research on MAP within Indigenous, northern, and rural communities. It will provide an updated summary of outcomes associated with these programs and identify best practices and potential areas for improvement. The importance of this review lies in its potential to inform policymakers, practitioners, and community leaders about effective strategies for addressing AUD in these underserved populations.

## 2. Methods

### 2.1. Study design

This scoping review adheres to the Preferred Reporting Items for Systematic Reviews and Meta-Analyses (PRISMA) guidelines to ensure a comprehensive and transparent review process. The review aims to evaluate the effectiveness and appropriateness of managed alcohol programs (MAP) in Indigenous, northern, rural and remote communities.

### 2.2. Information sources

A systematic search was employed on July 2, 2025 across multiple electronic databases to ensure a thorough literature search. Databases included PubMed, Embase, PsychINFO, and CINAHL. No data or language limits were used. Search terms were developed in order to identify a range of ways MAP could be characterized. Search terms included ‘managed alcohol programs’, ‘alcohol pours’, ‘northern’, ‘rural’, ‘remote’, ‘community’, ‘Indigenous’, ‘native American’, ‘Aboriginal’, ‘American Indian’. Next, we searched the grey literature by using the same search terms in Google Scholar and using iterative searching. Finally, the reference lists of included articles were searched to identify additional studies.

### 2.3. Study selection and eligibility criteria

The exclusion criteria were as follows: 1) did not contain full text of the article 2) were not in English 3) were not focused on programs that managed or regulated the consumption of alcohol 4) did not include participants who were experiencing homelessness and AUD and 5) did not involve Indigenous, northern, rural, or remote communities.

Two reviewers (MC and KM) independently screened all titles and abstracts identified through the search strategy and subsequently conducted independent full-text reviews of potentially eligible articles. Discrepancies at either stage were reviewed and resolved through discussion among all reviewers until consensus was reached. A standardized form was created to extract data in the following areas: [1] location, [2] study purpose, [3] study type and methodology, [4] sample size, [5] characteristics of the intervention and its implementation, [6] outcome measures and results, and [7] Indigenous, northern, and rural specific information identified in the study. Data extraction was completed by one reviewer (MC) who reviewed the extracted data for all included articles.

### 2.4. Reporting and transparency

The review findings are reported in accordance with the PRISMA guidelines, with a focus on clarity and transparency. A PRISMA flow diagram is provided to illustrate the study selection process, and a detailed summary of findings is presented. As this is a scoping review, we did not conduct a formal risk-of-bias assessment; however, most included studies were observational and descriptive in nature, with small samples and limited comparison groups, which may introduce selection, reporting, and confounding biases.

## 3. Results

**Fig 1:**
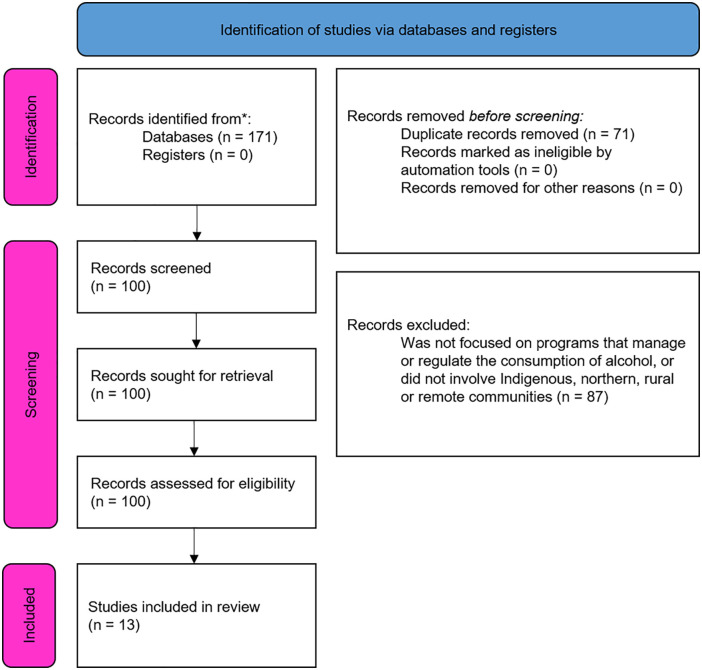
Flow diagram for identification of studies.

The literature search returned a total of 171 articles. Focused searching of gray literature and references found an additional 93 articles. After excluding duplicates, 100 studies were screened for title and abstract and full-text review. After title and abstract screening, 87 articles were eliminated due to irrelevance or if the full-text could not be found. 13 studies were included in the final review, as indicated in Fig 1 [20]. A list of these 13 studies can be found in Table 1. All articles in this review were based on residential programs.

**Table 1:** Studies included in this review.

Citation	Title	Country
Aboriginal Coalition to End Homelessness, 2018.	Indigenous pathways to health and well-being: Managed Alcohol Program feasibility study	Canada
Brocious et al., 2021	Managed alcohol: one community’s innovative response to risk management during COVID-19	United States
Brown et al., 2024	“Give me the reigns of taking care of myself with a home”: Healing environments in an Indigenous-led alcohol harm reduction program	Canada
Chow et al., 2017	Counting the cold ones: A comparison of methods measuring total alcohol consumption of managed alcohol program participants	Canada
Dumais et al., 2017	Come sit and be at home: A report on sharing circles with residents and staff from Ambrose Place	Canada
Motta-Ochoa et al., 2025	Barriers and facilitators to implementing a first managed alcohol program in Montreal, Canada	Canada
Pauly et al., 2016	Finding safety: a pilot study of managed alcohol program participants’ perception of housing and quality of life	Canada
Pauly et al., 2018	Community managed alcohol programs in Canada: Overview of key dimensions and implementation	Canada
Pauly et al., 2019	“There is a Place”: impacts of managed alcohol programs for people experiencing severe alcohol dependence and homelessness	Canada
Ramsperger et al., 2017	A selective literature review on managed alcohol programs and Indigenous healing methodologies	Canada
Stockwell et al., 2017	Does managing the consumption of people with severe alcohol dependence reduce harm? A comparison of participants in six Canadian managed alcohol programs with locally recruited controls	Canada
Thompson et al., 2025	Evaluation of a Residential Managed Alcohol Program for Aboriginal Peoples Experiencing Homelessness and Alcohol Dependence: Short-Term Impacts of an Australian Trial	Australia
Vallance et al., 2016	Do managed alcohol programs change patterns of alcohol consumption and reduce related harm? A pilot study	Canada

### 3.1. Description of MAPs

Established MAPs found within this review included programs located in supportive housing or shelters [17–19,21–27]. One MAP site emerged during the COVID-19 pandemic in short-term isolation or quarantine sites for vulnerable populations [28]. Eligibility criteria for MAPs typically included severe AUD, alcohol-related harms, homelessness, and unwillingness to enter or repeated unsuccessful experiences with other forms of AUD treatment. The type and amount of alcohol provided varied between programs; many programs dispense alcohol on an hourly basis with a maximum daily dosage, while other programs provide individualized rations of alcohol. Many of the programs have a physician or other healthcare professional determine the appropriate dosage of alcohol for clients. Most programs required clients to be on-site hourly to receive their dose. Policies vary regarding outside drinking between programs, ranging from no policies regarding outside drinking to forbidding outside drinking. Clients may have been refused alcohol or their dose may be altered if they appear intoxicated or if there is visible alcohol present in the client’s home. Other services are available for clients of MAP, including meals, medical and mental health services, case management, and other substance use disorder or harm reduction supports. Several programs have cultural supports specifically for Indigenous clients, including visits with Elders, support groups, smudging, drumming and singing [19,21,23,24,27].

The MAPs operating in Indigenous, northern, rural and remote communities that we identified in the literature are located within Canada [18,19,21–24,27], Australia [26], and the United States [28].

### 3.2. Themes emerged from the data

The findings of this review are categorized into four key themes, including: 1) Integration of Indigenous cultural knowledge and healing practices 2) Housing and finding “home” within MAP 3) Reduction of alcohol related harms 4) Barriers and facilitators to implementation.

#### 3.2.1. Integration of Indigenous cultural knowledge and healing practices.

Indigenous-led MAP are a limited but emerging area of programming, as four programs were identified within this review [21,23,24,26,28]. One study aimed to understand the feasibility of a MAP for Indigenous communities in Canada [29]. Several other MAP included in this review were not Indigenous-led, but primarily Indigenous people accessed its services [17,25,27].

Most of the MAP provided Indigenous-specific cultural supports, including opportunities to work with Elders and cultural practices, including traditional medicines and smudging, drumming, singing circles, traditional food, and language learning [17,23–25,27]. The integration of Indigenous cultural supports within MAP was consistently identified as important in contributing to participant well-being and healing. Many participants described their experience of being disconnected from Indigenous culture, as well as being disconnected from their families and communities [17,19]. Programs that were grounded in Indigenous values and that embedded cultural practices created environments where participants felt safer, respected, and more connected to family and community. Pauly and colleagues found that engagement with cultural practices can create opportunities for individuals to reconnect with their ancestral communities and cultural identity, which are factors that are often disrupted for Indigenous peoples with AUD and experiencing chronic homelessness [17,25]. Similarly, Thompson et al. suggest that incorporating Australian Aboriginal culture into harm reduction practices can provide meaningful pathways to stability and healing [26]. In several studies, participants reported that they found hope and opportunities for cultural and spiritual reconnection and identified this as particularly important in their healing journey [17]. Some programs offered the opportunity for participants to return to their home Indigenous communities for various reasons (cultural or community events, funerals, visiting family, etc.), which is essential in allowing individuals to reconnect with their identity and community [25–27].

The core values of MAP, which are based upon alcohol harm reduction principles, align with First Nations recovery principles. Smith-Bernardin et al. suggest that there is some contention about whether people should be able to participate in Indigenous cultural practices when they have been drinking or using drugs, as some Indigenous people believe that individuals need to abstain from substance use to participate [29]. A similar perspective emerged from a report on sharing circles with residents and staff of Ambrose Place, an Indigenous-led MAP in Edmonton, Alberta, Canada [23]. Ambrose Place follows a harm reduction philosophy, they accept residents where they are at, and their policy allows for alcohol consumption among residents; however, because heavy use of alcohol or drugs were not part of the way Indigenous peoples traditionally lived, and acknowledging the many historical and contemporary harms created by substance use, the underlying goal of Ambrose is to foster healing and, where possible, nurture a reduced dependence on all substances, including alcohol. These worldviews or perspectives are in contention with the core principle of MAP providing services without requiring abstinence.

There is emerging evidence that emphasizes the importance of Indigenous-led, self-determined MAP. There is a clear shift within the literature from previous MAP that offered Indigenous cultural supports but were led by non-Indigenous providers, to newer programs that are wholly designed and delivered by Indigenous communities. In Brown’s study on Indigenous led “culture-as-healing” models for SUD, participants described the empowerment of exercising autonomy over alcohol use within a program that simultaneously offered multiple entry points to cultural healing, such as land-based activities, ceremonies, and community connections [21]. A report on sharing circles with residents and staff from Ambrose Place (a supportive housing and MAP in Edmonton, Alberta, Canada) echoed these findings, noting that the presence of Elders, sharing circles, and cultural teachings fostered dignity, belonging, and recovery. Further, several feasibility and implementation studies included in this review identified cultural safety, flexibility, and Indigenous leadership as essential facilitators for successful program design and sustainability [29,30].

The literature demonstrates that Indigenous cultural supports within MAPs do more than enhance service acceptability. They actively reshape environments into spaces of healing, resilience, and self-determination. Strengthening connection to Indigenous identity via cultural supports providing participants with a sense of home, safety, and autonomy, while also strengthening the effectiveness of MAP in reducing harm and promoting holistic well-being.

#### 3.2.2. Housing and finding “home” within MAPs.

There were two distinct findings with respect to housing and security that were identified: first, participation in MAPs is associated with improved housing security and safety, and second, participants and staff often develop a sense of home and social and familial connection within the program itself.

All MAP participants prior to entering the program experienced chronic homelessness and had little to no sense of housing security. Access to supportive housing was identified as a key benefit of participation in a MAP by many participants and staff [25,26,30]. Across several studies included in this review, participation in a MAP was associated with retention of supportive housing and safer environments [18,27]. In a pilot study of a MAP in Thunder Bay, Ontario, Canada, MAP participants were more likely to retain housing than controls, and they scored significantly higher on several housing dimensions (including length of stay, safety, spaciousness, privacy, and overall quality) [25]. The stability provided by a stable and safe environment within the supportive housing as part of the MAP program allowed participants to shift their mindset from survival-based drinking to having autonomy and control over their alcohol use [21]. Some participants were able to cut down on their alcohol intake, which may be related to the stability and safety experienced within supportive housing [23,28].

In MAPs operating in Indigenous, northern, rural and remote communities, both participants and staff consistently emphasized the sense of home, belonging, and community that emerged within the programs themselves. Participants often described MAP as safe and non-judgemental spaces, particularly in comparison with their previous experiences on the streets, or in shelters, jails, or hospitals [17]. Participants used the words “home”, “family”, “safety”, “respect”, “trust”, and “hope” to characterize their experiences and relationships within a MAP in Thunder Bay, Ontario Canada [25]. MAP participants at Ambrose Place in Edmonton, Alberta, Canada echoed these experiences within their program, defining the program as home, safety, and community and likening their relationships they developed within the program (both with other residents and staff) to familial bonds [23]. MAP staff shared similar reflections about the importance of trust and belonging, suggesting that these relationships contributed positively to participants’ wellbeing and helped reshape their identity. For example, in a MAP in Montreal, Quebec, Canada, one staff member shared that as a result of the sense of community within the MAP, one resident no longer primarily identified as an Indigenous person experiencing homelessness and alcohol dependence, but instead as someone who belonged to a meaningful community within the MAP. It was identified as important that MAP must be designed as community-shaped, with policies that emphasize autonomy, dignity, and empowerment of participants [21].

#### 3.2.3. Reduction of alcohol-related harms.

MAP were consistently associated with reductions in alcohol-related harms across multiple studies. In particular, several studies report that participation in MAP is associated with decreased use of non-beverage alcohol (NBA). Participants reported a preference for program-provided beverage alcohol over NBA and reported significantly lower frequencies of NBA consumption compared to control groups [18,25]. Consumption of NBA is linked to a range of negative health effects that severely compromise the well-being of individuals with AUD. As such, one of the primary objectives of many MAPs across Canada and the world is to reduce participant consumption of NBA by providing easier access to beverage alcohol [25]. However, Chow found that participants report drinking more than twice the amount of alcohol administered within the program and often under-report the amount of drinking they do outside of the program. Thus, methods of measuring alcohol consumption within MAPs may underestimate the amount of beverage and NBA consumed by participants.

There are several health related benefits associated with MAP. Vallance found that MAP participants showed beneficial reductions in liver function tests (AST, ALT, and GGT levels) after entering the MAP. Participants in MAP experienced fewer alcohol-related health crises, with one study by Brocious reporting that no quarantined residents required hospitalization for alcohol withdrawal. Rates of acute service use (including hospitalizations, detox admissions, and emergency department presentations) were also substantially decreased after MAP entry [28]. Many participants were also able to access primary care and other medical services via the programs, which was identified as a significant positive outcome [17].

MAP also provide safer drinking environments that reduce the risks associated with street-based or other unsafe drinking environments [17]. These safer drinking environments served as spaces that encouraged healthier drinking patterns, as well as the opportunity to have choice and control over their alcohol use [21]. In addition to clinical and service-based outcomes, participants report experiencing fewer harms related to home life, unsafe environments, and legal issues [19,26].

#### 3.2.4. Barriers and facilitators to implementation of MAP in Indigenous, northern, rural and remote contexts.

A key barrier to implementation identified in several studies is resistance from local service providers, policymakers, and the community-at-large who are unfamiliar with harm reduction principles, particularly involving AUD [28]. MAP commonly face stigma during implementation, with some perceiving the programs as enabling alcohol use rather than reducing harm [27,28]. This resistance can result in delayed program development and instability or inefficiency of funding, which complicates program delivery [25]. However, providing education about alcohol withdrawal and alcohol harm reduction using peer reviewed literature and openly discussing potential associated challenges were identified as effective strategies to combat misconceptions about the alcohol harm reduction practices used in MAP [28].

Another barrier identified were systemic challenges associated with the delivery of health and social services in northern, rural and remote areas [27]. These communities often experience severe limitations in infrastructure and resources related to housing, social services, and healthcare. This was highlighted by Brocious where in Juneau, Alaska (a remote town accessible by boat or air) during the COVID-19 pandemic there were very limited hospital beds within the community hospital. In this case, the MAP emerged as a way to divert at-risk individuals and prevent hospitalizations related to COVID-19 and alcohol-related withdrawal, but infrastructure limitations still complicated the implementation of the MAP. Geographic isolation further complicates this issue, as it is difficult to facilitate access to consistent resources and staffing within the community [27].

The most commonly identified facilitator that supports the implementation of MAP in these contexts is that the program model is flexible and is driven by the needs of the community itself. Strong, trusting partnerships between program staff and local communities are important in ensuring program services are aligned with the needs of the community [18,21]. Flexibility in program delivery is essential for numerous reasons. Due to the unique health and social needs of individuals who access MAPs, there must be flexibility in structure and policy in order to meet the individual where they are at. Examples of flexibility can include: adjusting dispensing schedules, having take-away doses, low barrier admission criteria, rapid intake, offering mobile services, partnering with local services to help offer the service. This can be challenging for staff to manage, as highlighted by Motto-Ochoa, as it is difficult for staff to adapt to constantly changing environments. However, a flexible and accommodating approach helps build trust and positive relationships with participants, who often contrast this approach with more punitive systems that they have previously encountered [24]. Flexibility is also necessary to be able to operate in the unique contexts of Indigenous, northern, rural, and remote communities. Given the resource constraints often experienced by these communities, it was highlighted that programs must be able to adapt to and operate within these environments to succeed [28].

## 4. Discussion

This review set out to evaluate the characterize literature on MAP in Indigenous, northern, rural and remote contexts. Although the literature in this area remains limited, it appears there is a growing area of interest in the development, implementation, and efficacy of Indigenous-led MAP. There were a few studies identified that evaluated MAP operating in northern contexts, and there was only one study which described a MAP operating in a remote context [28]. Thus, there is a gap in understanding the complexities of MAP operating in northern, rural and remote contexts. Given the elevated rates of mental health, SUD, and alcohol-related harms in these communities, it is important to understand when MAP are an appropriate harm reduction method. It is evident that participation in MAP has several positive impacts on alcohol consumption and its associated harms, including decreased consumption of NBA, lower rates of alcohol-related hospitalizations, and reduced social and safety harms associated with illicit drinking [18,27]. While MAPs may reduce alcohol-related harms, participants may continue to experience risks associated with chronic heavy alcohol consumption.

Unlike other substances where harm reduction strategies can modify the method of consumption to lower risks alcohol’s cumulative effects on physical health cannot be mitigated in the same way [31]. Individuals living in Indigenous, northern, rural and remote communities already face disproportionately high risks of alcohol-related harms due to intersectional structural inequities, geographic isolation, and the impacts of historic and ongoing colonization. There are some concerns about potential harms associated with the continuation of long-term heavy alcohol use within the MAP model, and some suggest that there is a need to encourage reductions in alcohol volume while still respecting individual autonomy within MAP [31]. In addition, it has been demonstrated that MAP participants often underreport the amount of alcohol they drink outside of the program, and they report drinking more alcohol overall than what is administered within the program [22]. Special consideration about how to mitigate alcohol-related harms may need to be made in MAP operating in Indigenous, northern, rural, and remote communities, given that these communities already experience disproportionate alcohol-related harms.

A major theme that emerged was the strong sense of home and community that developed amongst participants and staff within the MAP. This environment characterized by safety, trust, and connection may be a key factor in the acceptability of MAP, particularly in Indigenous communities. Participants in Indigenous-led or culturally responsive MAP frequently described the environments they encountered within the program as places of belonging, care, and kinship [21]. These programs often extended beyond alcohol management to create spaces that fostered cultural safety, relationality, and connection to community. These features align with many Indigenous worldviews that prioritize relationality and connection to community. Métis Elder, author, and leader Maria Campbell describes the Cree/Métis principle of “wahkotowin”, which refers to the concept of kinship and interconnectedness, and emphasizes the reciprocal relationships and responsibilities between humans and all of creation [32]. This reflects a worldview of mutual respect, connection, and obligation across all forms of life. In several MAP, participants described their relationships with staff and other participants in familial terms, and there was a sense of responsibility and accountability featured within these relationships [23,24]. This is one poignant example of an Indigenous worldview that is aligned with the experiences of many Indigenous and non-Indigenous participants within MAP. The relational and community-oriented aspects of MAP appear to be compatible with Indigenous communities, such that local cultural values and community needs are at the forefront of the program.

Elders, community members, participants, and staff members shared that the core principles of MAP (which are rooted in alcohol harm reduction principles) align with many Indigenous approaches to healing from SUD [24,26]. While Indigenous worldviews are diverse across communities, many share a common holistic perspective on health, putting importance on the balance of mind, body, and spirit. MAP reflect a similar philosophy by providing support for the individual in many different aspects of life [17]. In many programs, in addition to receiving regular access to beverage alcohol, individuals can access shelter, mental and physical health care, counselling, and cultural supports through the program. In this sense, all needs of the individual are being attended to and supported in order to promote overall health and well-being.

It emerged that some Indigenous peoples regard alcohol as possessing a harmful spiritual presence that undermines Indigenous ways of life, with addiction seen as a force that disrupts traditional cultural systems [33]. Smith-Bernardin et al. [29] shared a teaching from Elder Dila Provost Houle of the Piikani First Nation. According to this teaching, substance use can disrupt an individual’s connection to their spirit, and healing involves supporting the restoration of spiritual, cultural, and relational connections. A similar perspective is shared within the MAP at Ambrose Place in Edmonton, Alberta, Canada, as heavy alcohol or drug use were not part of the way Indigenous people traditionally lived and in recognition of the many historical and contemporary harms affecting communities due to substance use, they differentiate their primary goal from some other MAP in Canada and aim to eventually reduce alcohol use [23]. This perspective is in contention with the core, guiding principle of MAP which do not require abstinence as a goal. It is important to recognize the incredible diversity of Indigenous communities, and that perspectives on abstinence and alcohol harm reduction may vary between communities. In order to ensure MAP are culturally safe and appropriate, there must be involvement from the local Indigenous community to ensure their unique perspective and needs are met by the program.

Indigenous-led MAP and the integration of cultural supports within AUD services may play a critical role in supporting healing and recovery for Indigenous peoples with AUD. Within this review, access to cultural supports within MAP was identified to be essential in facilitating healing, recovery, and reconnection to identity [17,19,23,24]. Research has shown that Indigenous-led programs for SUD that are grounded in traditional cultural practices promote cultural identity and connection, which are essential in healing from SUD [34,35]. Culturally integrated substance use programs for Indigenous peoples are associated with improvements in wellness and reductions in substance use [36]. An Indigenous community in Alkali Lake, BC, employed traditional Indigenous healing methodologies and healers to help revive traditional dances, ceremonies, and spiritual practices as methods of re-connecting to culture. Through these measures, the community decreased its rate of alcohol abuse from 95% to 5% in 10 years [36]. Thus, cultural supports can significantly support Indigenous peoples healing from AUD and other SUD. These findings suggest that the success of MAP in Indigenous communities depends on the extent to which programs are based upon cultural knowledge, community needs, and self-determination and self-governance.

### 4.1. Limitations

Several limitations of scoping reviews should be considered when interpreting these findings. Consistent with the purpose of scoping reviews, this review aimed to map and characterize the existing literature, rather than critically evaluate the quality of the evidence. This approach limits the ability to determine the strength of the reported findings. As well, although a comprehensive search strategy was used, it is possible that relevant studies were missed or excluded for reasons of text availability or publication language. Finally, as most of the included studies originated in Canada, the results may not be generalizable to all geographic or health system contexts.

## 5. Conclusion

This scoping review provides a summary of the current research on MAP for individuals with severe AUD in Indigenous, northern, rural, and remote communities. The current evidence suggests that MAP, when tailored to the specific cultural, social, and environmental contexts of these communities, can play a crucial role in supporting individuals with AUD and promoting community well-being. The review identifies several key factors contributing to the success of MAP, including peer and community involvement, culturally appropriate supports, and integrated health and supportive services.

While research on MAP continues to grow, there remains a lack of focused investigation into their effectiveness and the availability of AUD-related services particularly in northern, rural and remote contexts. There remains limited data on how MAP function in geographically isolated areas where access to services, housing, and healthcare is often limited. This lack of evidence poses a challenge for informing policy and programming in areas where alcohol-related harms disproportionately impact communities.

Even though there is this gap, an emerging area of focus should be on Indigenous-led MAP that are created by Indigenous peoples, for Indigenous peoples. These self-determined programs offer transformational cultural support and relational models of care for Indigenous peoples with AUD. These programs do not solely offer a harm reduction model to manage severe AUD, but do so in a way that emphasizes community, connection to land, and wholistic wellness.

Future research should prioritize the evaluation and support of MAP designed by Indigenous communities, particularly those in northern, rural, and remote settings. It is critical to champion culturally safe, sustainable programs for alcohol harm reduction.

## Supporting information

S1 Checklist Preferred reporting items for systematic reviews and meta-analyses extension for scoping reviews (PRISMA-ScR) checklist. (PDF)

S1 Table Details of studies included in this review. (XLSX)
