# Supplementary material for: Managed alcohol programs for Indigenous Peoples in Canada: A scoping review
Source: PLOS Ment Health. 2026 Jul 14;3(7):e0000651. doi: 10.1371/journal.pmen.0000651 (PMC13367720; doi:10.1371/journal.pmen.0000651)
Supplement: Attachment — Submitted filename: Response to reviewer.docx [file pmen.0000651.s003.docx]

**Response to reviewer**

**We note that this manuscript is a scoping review; our author guidelines require that you upload a PRISMA-ScR checklist as supporting information. Information about the PRISMA guidance and blank checklists can be found here:** [**https://www.prisma-statement.org/scoping**](https://www.prisma-statement.org/scoping)**).**

Thank you, we have now added the checklist and a Table 1
