# Supplementary material for: Managed alcohol programs for Indigenous Peoples in Canada: A scoping review
Source: PLOS Ment Health. 2026 Jul 14;3(7):e0000651. doi: 10.1371/journal.pmen.0000651 (PMC13367720; doi:10.1371/journal.pmen.0000651)
Supplement: Attachment — Submitted filename: Response to Reviewers_10Jun2026.docx [file pmen.0000651.s004.docx]

**Reviewer #1: Overall comment: This study presents a timely investigation of an important harm reduction intervention, managed alcohol programs. As a personal note, I am quite interested in the thematic results and applaud the authors for their undertaking. My comments are offered in the spirit of strengthening the manuscript.

Major comments
 1. Introduction, line 56: Referring to MAP alcohol dispensation as “controlled” prompts me to wonder if the potential carceral politics of such programs will be addressed in the discussion?**

Thank you, the use of the term "controlled" refers to the structured and scheduled dispensing of alcohol within MAPs to support safety and harm reduction objectives, rather than to mechanisms of surveillance or coercion. While questions regarding autonomy and the potential carceral dimensions of service delivery are important, they are beyond the scope of the current study. We adjusted the language to standardized instead (line 56)

**2. In addition to more complete information in the supplemental tables, an in-article table with basic information regarding the 13 included studies would be helpful for the reader’s reference.**

Thank you for your suggestion, this table has been added.

**3. I would suggest some revision such that specific information regarding included publications is included in the Results section, and broader implications drawn from these findings is included in the Discussion. I point out a few examples below, but I would suggest the authors read through these two sections with a critical eye.
 a. Results, lines 176-181: The authors offer an important perspective on MAP cultural appropriateness here; I wonder if these thoughts are better suited to the Discussion.**

Thank you, this was moved to the discussion

**Possibly also the paragraph lines 195-199?**

Thank you, I added an example in the discussion (line 339)

**b. Discussion, lines 347-357: This specific quote may fit better in the Results?**

Thank you, we removed the comment, and summarized the finding. Line 365
 **Minor comments
 1. Introduction, lines 33-34: I suspect that alcohol dependence refers to physical dependence, indicating a subset of people with AUD? A bit clarification about the distinction between 400 million people meeting criteria for AUD and 209 million people with alcohol dependence would be appreciated.**

Thank you, this was a mistake. We are not distinguishing physical dependence and other. We are only talking about AUD here. (line 33)

**2. Method, lines 93-95: I understand that the two reviewers screened each article independently, and then re-screened all articles screened by the other reviewer? Or did the reviewers screen independently and review conflicts with the other reviewer?**

Thank you, this was clarified (line 93)

**3. Method, lines 99-100: I believe if one reviewer completed all data extraction, the specification that they did so independently is not necessary.**

Thank you for your comment, this has been removed.

**4. Figure 1: Would it be possible to remove the paragraph markers?**

This was removed

**5. Figure 1: I understand from the figure that no records were removed at the title and abstract screening phase?**

Yes 87 were removed line 115 and figure 1

**6. Figure 1: What does “reports of included studies” refer to?**

That was removed.

**7. Results, line 117: Could the authors clarify that all MAPs included in this review were residential programs? I believe this is the case based on this introductory sentence.**

This has been clarified.

**8. Results, line 120: I believe the authors intended to indicate “homelessness” as a criterion for MAP enrollment.**

Thank you, this has been corrected.

**9. Results, line 121: I would caution the authors to be wary of stigmatizing language such as “failure.”**

Thank you, this wording has been changed.

**10. Results, lines 142-143: The authors report that a few studies aimed to understand MAP feasibility for Indigenous communities in Canada, but cite only one study.**

Thank you, this has been clarified to match citing one study,

**11. Results, lines 281-294: Could the authors give an example of the program flexibility from an included publication?**

Thank you, we added more context (line 295)

**12. Discussion, line 296: As a scoping review without an assessment of study bias, I’m not sure that “evaluate” is the appropriate verb.**

Thank you, this has been changed to “characterize”.

**13. Discussion, line 307: I’m a bit confused by this sentence. The benefits associated with MAP participation sustained alcohol use? Is the point that MAP participation itself sustains alcohol use? And how do the authors define “heavy” use?**

Thank you, this was reworded on line 317.

**Reviewer #2: The manuscript is a well-conducted scoping review expressed clearly and succinctly. Methods are transparent and the inferences made from the available data are warranted and replicable. However, a discission on the limitations of the scoping review process is absent and must therefore be added before publication can be authorized.**

Thank you for your comments. A limitations section has been added to address the limitations of scoping reviews.
