# Supplementary material for: Managed alcohol programs for Indigenous Peoples in Canada: A scoping review
Source: PLOS Ment Health. 2026 Jul 14;3(7):e0000651. doi: 10.1371/journal.pmen.0000651 (PMC13367720; doi:10.1371/journal.pmen.0000651)
Supplement: Peer review history

# Author comment: Author response to Decision Letter 0

**DOI:** 10.1371/journal.pmen.0000651.r001

11 Mar 2026

Attachment Submitted filename: Response to reviewer.docx

# Peer review: Decision Letter 0

**DOI:** 10.1371/journal.pmen.0000651.r002

**Authors:** Karli Montague-Cardoso

10 Jun 2026

PMEN-D-26-00130

Managed alcohol programs for Indigenous Peoples in Canada: A scoping review

PLOS Mental Health

Dear Dr. Marsh,

Thank you for submitting your manuscript to PLOS Mental Health. I am sorry for the delay in reaching a decision, which was due to difficulties securing reviewers. After careful consideration of the reviewer reports, we feel that your manuscript has merit but does not fully meet PLOS Mental Health’s publication criteria as it currently stands. Therefore, we invite you to submit a revised version of the manuscript that addresses the points raised during the review process. You can find all of the points that need to be addressed below and in the attached document.

Please submit your revised manuscript by Jul 08 2026 11:59PM. If you will need more time than this to complete your revisions, please reply to this message or contact the journal office at mentalhealth@plos.org. When you’re ready to submit your revision, log on to https://www.editorialmanager.com/pmen/ and select the ‘Submissions Needing Revision’ folder to locate your manuscript file.

Please include the following items when submitting your revised manuscript:

- A letter that responds to each point raised by the editor and reviewer(s). You should upload this letter as a separate file labeled ‘Response to Reviewers’.
- A marked-up copy of your manuscript that highlights changes made to the original version. You should upload this as a separate file labeled ‘Revised Manuscript with Track Changes’.
- An unmarked version of your revised paper without tracked changes. You should upload this as a separate file labeled ‘Manuscript’.

Guidelines for resubmitting your figure files are available below the reviewer comments at the end of this letter.

As the corresponding author, your ORCID iD is verified in the submission system and will appear in the published article. PLOS supports the use of ORCID, and we encourage all coauthors to register for an ORCID iD and use it as well. Please encourage your coauthors to verify their ORCID iD within the submission system before final acceptance, as unverified ORCID iDs will not appear in the published article. *Only*  the individual author can complete the verification step; PLOS staff *cannot*  verify ORCID iDs on behalf of authors.

We look forward to receiving your revised manuscript.

Kind regards,

Karli Montague-Cardoso

Staff Editor

PLOS Mental Health

**Journal Requirements:**

1. Please amend your detailed Financial Disclosure statement. This is published with the article. It must therefore be completed in full sentences and contain the exact wording you wish to be published.

i. Please clarify all sources of financial support for your study. List the grants, grant numbers, and organizations that funded your study, including funding received from your institution. Please note that suppliers of material support, including research materials, should be recognized in the Acknowledgements section rather than in the Financial Disclosure.

ii. State the initials, alongside each funding source, of each author to receive each grant. For example: "This work was supported by the National Institutes of Health (####### to AM; ###### to CJ) and the National Science Foundation (###### to AM)."

iii. State what role the funders took in the study. If the funders had no role in your study, please state: “The funders had no role in study design, data collection and analysis, decision to publish, or preparation of the manuscript.”

iv. If any authors received a salary from any of your funders, please state which authors and which funders.

2. In the online submission form, you indicated that “Data can be obtained upon reasonable request”.

All PLOS journals now require all data underlying the findings described in their manuscript to be freely available to other researchers, either

1. In a public repository,

2. Within the manuscript itself, or

3. Uploaded as supplementary information.

This policy applies to all data except where public deposition would breach compliance with the protocol approved by your research ethics board. If your data cannot be made publicly available for ethical or legal reasons (e.g., public availability would compromise patient privacy), please explain your reasons by return email and your exemption request will be escalated to the editor for approval. Your exemption request will be handled independently and will not hold up the peer review process, but will need to be resolved should your manuscript be accepted for publication. One of the Editorial team will then be in touch if there are any issues.

3. We have noticed that you have uploaded Supporting Information files, but you have not included a list of legends. Please add a full list of legends for your Supporting Information files after the references list.

If the reviewer comments include a recommendation to cite specific previously published works, please review and evaluate these publications to determine whether they are relevant and should be cited. There is no requirement to cite these works unless the editor has indicated otherwise.

Please review your reference list to ensure that it is complete and correct. If you have cited papers that have been retracted, please include the rationale for doing so in the manuscript text, or remove these references and replace them with relevant current references. Any changes to the reference list should be mentioned in the rebuttal letter that accompanies your revised manuscript. If you need to cite a retracted article, indicate the article’s retracted status in the References list and also include a citation and full reference for the retraction notice.

**Additional Editor Comments (if provided):**

[Note: HTML markup is below. Please do not edit.]

Reviewers’ comments:

Reviewer’s Responses to Questions

**Comments to the Author**

1. Does this manuscript meet PLOS Mental Health’s publication criteria? Is the manuscript technically sound, and do the data support the conclusions? The manuscript must describe methodologically and ethically rigorous research with conclusions that are appropriately drawn based on the data presented.

Reviewer #1: Yes

Reviewer #2: Yes

**********

2. Has the statistical analysis been performed appropriately and rigorously?

Reviewer #1: N/A

Reviewer #2: N/A

**********

3. Have the authors made all data underlying the findings in their manuscript fully available (please refer to the Data Availability Statement at the start of the manuscript PDF file)?

The PLOS Data policy requires authors to make all data underlying the findings described in their manuscript fully available without restriction, with rare exception. The data should be provided as part of the manuscript or its supporting information, or deposited to a public repository. For example, in addition to summary statistics, the data points behind means, medians and variance measures should be available. If there are restrictions on publicly sharing data—e.g. participant privacy or use of data from a third party—those must be specified.

Reviewer #1: Yes

Reviewer #2: Yes

**********

4. Is the manuscript presented in an intelligible fashion and written in standard English?

PLOS Mental Health does not copyedit accepted manuscripts, so the language in submitted articles must be clear, correct, and unambiguous. Any typographical or grammatical errors should be corrected at revision, so please note any specific errors here.

Reviewer #1: Yes

Reviewer #2: Yes

**********

5. Review Comments to the Author

Please use the space provided to explain your answers to the questions above. You may also include additional comments for the author, including concerns about dual publication, research ethics, or publication ethics. (Please upload your review as an attachment if it exceeds 20,000 characters)

**Reviewer #1** : Overall comment: This study presents a timely investigation of an important harm reduction intervention, managed alcohol programs. As a personal note, I am quite interested in the thematic results and applaud the authors for their undertaking. My comments are offered in the spirit of strengthening the manuscript.

Major comments

1. Introduction, line 56: Referring to MAP alcohol dispensation as “controlled” prompts me to wonder if the potential carceral politics of such programs will be addressed in the discussion?

2. In addition to more complete information in the supplemental tables, an in-article table with basic information regarding the 13 included studies would be helpful for the reader’s reference.

3. I would suggest some revision such that specific information regarding included publications is included in the Results section, and broader implications drawn from these findings is included in the Discussion. I point out a few examples below, but I would suggest the authors read through these two sections with a critical eye.

a. Results, lines 176-181: The authors offer an important perspective on MAP cultural appropriateness here; I wonder if these thoughts are better suited to the Discussion. Possibly also the paragraph lines 195-199?

b. Discussion, lines 347-357: This specific quote may fit better in the Results?

Minor comments

1. Introduction, lines 33-34: I suspect that alcohol dependence refers to physical dependence, indicating a subset of people with AUD? A bit clarification about the distinction between 400 million people meeting criteria for AUD and 209 million people with alcohol dependence would be appreciated.

2. Method, lines 93-95: I understand that the two reviewers screened each article independently, and then re-screened all articles screened by the other reviewer? Or did the reviewers screen independently and review conflicts with the other reviewer?

3. Method, lines 99-100: I believe if one reviewer completed all data extraction, the specification that they did so independently is not necessary.

4. Figure 1: Would it be possible to remove the paragraph markers?

5. Figure 1: I understand from the figure that no records were removed at the title and abstract screening phase?

6. Figure 1: What does “reports of included studies” refer to?

7. Results, line 117: Could the authors clarify that all MAPs included in this review were residential programs? I believe this is the case based on this introductory sentence.

8. Results, line 120: I believe the authors intended to indicate “homelessness” as a criterion for MAP enrollment.

9. Results, line 121: I would caution the authors to be wary of stigmatizing language such as “failure.”

10. Results, lines 142-143: The authors report that a few studies aimed to understand MAP feasibility for Indigenous communities in Canada, but cite only one study.

11. Results, lines 281-294: Could the authors give an example of the program flexibility from an included publication?

12. Discussion, line 296: As a scoping review without an assessment of study bias, I’m not sure that “evaluate” is the appropriate verb.

13. Discussion, line 307: I’m a bit confused by this sentence. The benefits associated with MAP participation sustained alcohol use? Is the point that MAP participation itself sustains alcohol use? And how do the authors define “heavy” use?

**Reviewer #2:**  The manuscript is a well-conducted scoping review expressed clearly and succinctly. Methods are transparent and the inferences made from the available data are warranted and replicable. However, a discission on the limitations of the scoping review process is absent and must therefore be added before publication can be authorized.

**********

6. PLOS authors have the option to publish the peer review history of their article (what does this mean?). If published, this will include your full peer review and any attached files.

**Do you want your identity to be public for this peer review?**  If you choose “no”, your identity will remain anonymous but your review may still be made public.

For information about this choice, including consent withdrawal, please see our Privacy Policy.

Reviewer #1: No

Reviewer #2: No

**********

[NOTE: If reviewer comments were submitted as an attachment file, they will be attached to this email and accessible via the submission site. Please log into your account, locate the manuscript record, and check for the action link "View Attachments". If this link does not appear, there are no attachment files.]

**Figure Resubmissions:**

While revising your submission, we strongly recommend that you use PLOS’s NAAS tool (https://ngplosjournals.pagemajik.ai/artanalysis) to test your figure files. NAAS can convert your figure files to the TIFF file type and meet basic requirements (such as print size, resolution), or provide you with a report on issues that do not meet our requirements and that NAAS cannot fix.

After uploading your figures to PLOS’s NAAS tool - https://ngplosjournals.pagemajik.ai/artanalysis, NAAS will process the files provided and display the results in the "Uploaded Files" section of the page as the processing is complete. If the uploaded figures meet our requirements (or NAAS is able to fix the files to meet our requirements), the figure will be marked as "fixed" above. If NAAS is unable to fix the files, a red "failed" label will appear above. When NAAS has confirmed that the figure files meet our requirements, please download the file via the download option, and include these NAAS processed figure files when submitting your revised manuscript.

# Author comment: Author response to Decision Letter 1

**DOI:** 10.1371/journal.pmen.0000651.r003

11 Jun 2026

Attachment Submitted filename: Response to Reviewers_10Jun2026.docx

# Editor report: Decision Letter 1

**DOI:** 10.1371/journal.pmen.0000651.r004

**Authors:** Karli Montague-Cardoso, Karli Montague-Cardoso

17 Jun 2026

Managed alcohol programs for Indigenous Peoples in Canada: A scoping review

PMEN-D-26-00130R1

Dear Dr. Marsh,

We are pleased to inform you that your manuscript ‘Managed alcohol programs for Indigenous Peoples in Canada: A scoping review’ has been provisionally accepted for publication in PLOS Mental Health.

Before your manuscript can be formally accepted you will need to complete some formatting changes, which you will receive in a follow up email. A member of our team will be in touch with a set of requests.

Please note that your manuscript will not be scheduled for publication until you have made the required changes, so a swift response is appreciated.

IMPORTANT: The editorial review process is now complete. PLOS will only permit corrections to spelling, formatting or significant scientific errors from this point onwards. Requests for major changes, or any which affect the scientific understanding of your work, will cause delays to the publication date of your manuscript.

If your institution or institutions have a press office, please notify them about your upcoming paper to help maximize its impact. If they’ll be preparing press materials, please inform our press team as soon as possible -- no later than 48 hours after receiving the formal acceptance. Your manuscript will remain under strict press embargo until 2 pm Eastern Time on the date of publication. For more information, please contact mentalhealth@plos.org.

Thank you again for supporting Open Access publishing; we are looking forward to publishing your work in PLOS Mental Health.

Best regards,

Karli Montague-Cardoso

Staff Editor

PLOS Mental Health

***********************************************************

Reviewer Comments (if any, and for reference):
